# Determining the research status and coronavirus anxiety scores of academics during the flexible working arrangements initiated after the COVID-19 pandemic

**DOI:** 10.1016/j.jtumed.2021.01.005

**Published:** 2021-02-09

**Authors:** Deniz Akyildiz, Serife Durna

**Affiliations:** a Kahramanmaras Sutcu Imam University, Faculty of Health Sciences, Division of Midwifery, Kahramanmaras, Turkey; b Kahramanmaras Sutcu Imam University, School of Foreign Languages, Kahramanmaras, Turkey

**Keywords:** أكاديمي, القلق, فيروس كورونا, كوفيد-١, تركيا, Academician, Anxiety, Coronavirus, COVID-19, Turkey

## Abstract

**Objectives:**

This study aims to determine the effect of flexible work arrangements (FWA) initiated in Turkey due to the COVID-19 pandemic on research status of academics and their coronavirus anxiety scores.

**Methods:**

In this cross-sectional study, 290 academicians participated and completed the questionnaire. The descriptive (by response frequency) and inferential statistics (chi-square, student's t, and Anova tests) were performed for advanced data analysis.

**Results:**

We found that a majority of the academics (71%) did not conduct academic research after the outbreak of the COVID-19 pandemic and academic research was largely negatively affected (67.2%). Furthermore, women (53.9%) and those with higher Coronavirus Anxiety Scale (CAS) scores had low research output. Additionally, women (54.9%), the participants working in health faculties (52.8%) and those with a high CAS score were adversely affected (*p* < 0.001). Women, those working in healthcare faculties, and the younger participants had the highest CAS score (*p* < 0.001).

**Conclusions:**

The results of this study provide important data on the effects of the FWA implemented during the COVID-19 pandemic on academic research status and the coronavirus anxiety scores of academics.

## الملخص

**أهداف البحث:**

تهدف هذه الدراسة إلى تحديد تأثير الفيروس التاجي على حالة البحوث الأكاديمية ودرجات القلق خلال ترتيبات العمل المرنة بعد جائحة كوفيد-١٩ في تركيا.

**طرق البحث:**

شارك ٢٩٠ أكاديميا أكملوا الاستبانة في هذه الدراسة الشاملة لعدد من القطاعات. تم تنفيذ الإحصاءات الوصفية (حسب تردد الاستجابة) والإحصائيات الاستدلالية لتحليل البيانات المتقدم.

**النتائج:**

وجدنا أن معظم الأكاديميين (٧١٪) لم يجروا البحوث الأكاديمية بعد جائحة كوفيد-١٩وتأثرت معظم البحوث التي أجراها الأكاديميون سلبا (٦٧.٢٪). وعلاوة على ذلك، انخفاض النتاج البحثي من البحوث الأكاديمية التي أجرتها الأكاديميات (٥٣.٩٪) وللذين لديهم معدل أعلى لمقياس كورونا للقلق. وبالإضافة إلى ذلك، تأثرت النساء (٥٤.٩٪)، والمشاركات العاملات في الكليات الصحية (٥٢.٨٪). وسجلت النساء والعاملات في الكليات الصحية، والمشاركات الأصغر سنا درجات أعلى لمقياس كورونا للقلق.

**الاستنتاجات:**

تقدم نتائج هذه الدراسة بيانات مهمة عن آثار اللوائح خلال فترة عمل الترتيبات العمل المرنة بعد جائحة كوفيد-١٩على حالة البحوث الأكاديمية، ودرجات القلق من الفيروس التاجي للأكاديميين.

## Introduction

In December 2019, a new type of coronavirus (COVID-19) emerged as a viral pandemic in Wuhan, China's Hubei province.^1^ The virus had spread almost all over the world by March 23, 2020, and infected more than 294.110 people in 187 countries, causing the death of 12.944 people.^2^ Most of the countries affected by the pandemic have taken various measures to slow the transmission, such as closing workplaces, hygiene regulations, social distancing practices, closing schools and universities, and flexible working arrangements (FWA).^3^^,^^4^

In Turkey, the first cases of COVID-19 were detected on March 11, 2020. Following this, curfews were imposed during certain periods between April and June in some major provinces, including the province of Kahramanmaras. The Turkey Higher Education Council has initiated the application of distance education in all universities in the country and FWA was launched for academics March 22, 2020 onwards. Following the publication of a circular regarding FWA in Turkey, remote work for academics and flexible working methods such as interleaving were initiated. It was accepted that academicians could not leave their city of residence without the permission of their superiors, personnel whose services were needed had to return to their duty as soon as they were called, and individuals were allowed to work from home.

This arrangement is referred to through different terms such as FWA, remote working, working from home, and teleworking.^4^ FWA to prevent the spread of COVID-19 are expected to have short and long-term positive or negative effects on people's working lives.^3^ On one hand, positive effects of FWA have been reported such as time spent on the road or in traffic, dead times in the workplace and increased productivity, spending more time with their families, dealing with children, and allocating time for distance education of children.^5^ On the other, negative effects of FWA such as not being able to meet face to face, lack of adequate support and infrastructure facilities, technical problems, increased home and child care workload have been identified.^6^^,^^7^ In a qualitative study carried out in Turkey during the COVID-19 pandemic, it was determined that FWA negatively affects work efficiency due to reasons such as change in the work environment, the need for information technology infrastructure, stretching and elongation of working time, the difficulty of holding meetings in homes with children, and increased workload.^8^ According to these findings, it can be said that the effects of FWA during the pandemic are varied.

In addition to efforts to prevent the transmission of the virus, it is important to note that the pandemic also affects the psychological health of people.^9^ Studies conducted in India, Turkey and China revealed that anxiety was one of the most common psychological problems reported at the beginning of the pandemic.^10^, ^11^, ^12^ The high anxiety detected during the course of the pandemic is an important situation that needs to be evaluated and controlled. Previous studies show that high anxiety reduces students' academic performance.^13^^,^^14^ The existing research also shows that during the COVID-19 epidemic, women had higher anxiety levels than men,^15^, ^16^, ^17^, ^18^, ^19^ and the productivity and scientific results of female academicians were negatively affected during the pandemic process.^20^ Since high anxiety level can affect academic success, coronavirus anxiety that develops during the pandemic process may also affect academic research.

It is important to determine the effects of the pandemic on academicians who are expected to produce scientific knowledge because scientific research forms the basis of the developments in science and technology.^21^ In particular, it is crucial to reduce the disease and its effects using the scientific knowledge revealed by academic studies conducted during the pandemic. Thus, it is essential to determine the effects of the current situation on academicians. This study aims to determine the effect of FWA on academicians and their academic research status and the coronavirus anxiety scores during the FWA initiated due to the COVID-19 pandemic in Turkey. The research questions are the following: (1) What is the status of academics in terms of conducting academic research during the post-pandemic FWA process? (2) How was academic research affected in this process? (3) What influenced their academic research and (4) how did they conduct academic research? (5) What are the CAS levels of academics during this period? (6) What are the factors related to CAS?

## Materials and Methods

### Study design

The study has a cross-sectional design. It was conducted from July 2020 to September 2020 during the COVID-19 pandemic.

### Study area

The study was conducted in Kahramanmaras Sutcu Imam University (KSU), located in Kahramanmaras centre in Turkey.

### Sample population

This study was conducted in the second semester of the 2019–2020 academic year, when FWA was fully implemented due to the COVID-19 pandemic. FWA at KSU includes a process that allows academics to work from home. In this study arrangement, people are allowed to work from home but academicians cannot leave the city they live in without the permission of their administrators and the personnel whose services are required must return to their duties as soon as they are called.

The research population consisted of 1042 academicians working at KSU. The sample of the research was composed of 290 academicians who were included in the study between July 2020 and September 2020, during the data collection phase. In total, 1042 academicians were evaluated for the study. Of these, 14 were eliminated because they could not speak or understand Turkish, 9 of them did not agree to participate in the research, and 729 did not reply to the e-mail sent. Inclusion criteria for the study were: working as an academician at KSU, being able to speak and understand Turkish, having internet access, and agreeing to participate in the research.

### Data collection

All academicians working at the university between July 2020 and September 2020 were invited to participate in the study via e-mail, using the e-mail addresses available on the university website. The purpose of the research and the link of the questionnaire forms created through Google Forms were sent to the participants by e-mail. Research data were collected online. Before starting the survey, all participants were informed of the written consent forms sent to their e-mails. Written informed consent was obtained by e-mail from the participants who accepted the invitation to participate in the study and completed the questionnaire.

### Instruments

For the collection of research data, a 26-questions questionnaire form created by the researchers based on existing literature and a 5-question Coronavirus Anxiety Scale-Short Form were used.^3^^,^^16^^,^^29^ The questionnaire form developed by the researchers was presented to 10 faculty members working in different fields for their expert opinion. Following the evaluation made by the faculty members, the data collection forms were evaluated using the Lawshe technique. The Content Validity Criterion (CVI) value for 10 experts in the questionnaire was determined as 0.636. The Content Validity Index (CVI) of the 26 questions was calculated as 0.910. The fact that the CGI value obtained is greater than the CVI value (0.910 > 0.636) indicates that the content validity of the questionnaire form items is statistically significant. It was concluded that all questionnaire questions prepared based on this analysis were appropriate for use in the study. A pilot application was conducted through an online questionnaire administered to 5 participants who represented the target population of survey study. The results of the pilot test were not included in the final results. The research questions consisted of 3 parts.

*Part 1. Demographic information:* It consisted of 12 questions to evaluate the demographic information of the participants such as age, gender, marital status, graduation level, title, and the faculty where they work.

*Part 2. Evaluation of academic research status:* This consisted of 14 questions to evaluate the academic research status of academicians. It included: (1) questions (positive or negative) about whether they have done academic research during the FWA process (yes or no) and (2) questions about how their academic research is affected. In addition, the questionnaire focused on factors affecting the studies positively and negatively, whether they wanted FWA to continue, the number and types of academic studies in this process, and whether they worked on COVID-19.

*Part 3. Determining coronavirus anxiety scores:* The Coronavirus Anxiety Scale (CAS) was used to determine the COVID-19 anxiety levels of the participants. The scale was developed by Lee (2020) and adapted to Turkish by Bicer et al. (2020) by conducting a Turkish validity and reliability study.^22^^,^^23^ It is a Likert-type scale consisting of 5 items, scored between 0 and 4. Bicer et al. (2020) found that the Cronbach Alpha reliability coefficient of the scale was 0.832, while it was 0.948 in our study.

### Dependent and independent variables

The dependent variables of the study are the academic research status of academicians (‘conducting academic research’ and ‘how academic research was affected’) and their CAS. Independent variables were determined as gender, age, marital status, having children, type of faculty, and title.

### Data analysis

The Statistical Package for the Social Sciences Version 21.0 (SPSS Inc., IL, USA) was used for the data analysis. The academician's demographic, academic studies, and satisfaction with the FWA were evaluated with descriptive statistics. The t-test, chi-square, and Anova tests were used to compare the how academic studies were affected and the CAS score to the various variables. The Alpha value of the CAS score was calculated using reliability analysis.

## Results

### Demographics of study sample

The response rate was 27.8% (n = 290 out of a total of 1042 academicians). The majority of 290 academicians were male (56.2%), married (74.1%), and Dr. Lecturer (48.6%). Table 1 summarises the descriptive statistics of participants’ demographic characteristics.

**Table 1:** Demographic characteristics of academicians (N=290).

Variables	n	%
**Age**		
29-34	105	36.2
35-44	101	34.8
45-54	66	23.8
55-64	15	5.2
**Gender**		
Female	127	43.8
Male	163	56.2
**Marital status**		
Single	75	25.9
Married	215	74.1
**Having a child**		
Yes	220	75.9
No	70	24.1
**Title**		
Prof. Dr.	22	7.6
Assoc. Dr.	24	8.3
Dr. Lecturer	141	48.6
Instructor	92	31.7
Research Assistant	11	3.8
**Faculty Type**		
Health^a^	107	36.9
Others	183	63.1
**Faculty Name**		
Faculty of Dentistry	16	5.5
Faculty of Education	24	8.3
Faculty of Science and Literature	25	8.6
Faculty of Arts	7	2.4
Faculty of Economics and Administrative Sciences	8	2.8
Faculty of Theology	10	3.4
Faculty of Engineering and Architecture	7	2.4
Faculty of Health Sciences	22	7.9
Medical School	42	14.4
Faculty of Agriculture	16	5.5
School of Foreign Languages	11	3.7
Health Services Vocational School	27	9.2
Vocational School of Social Sciences	47	16.2
Vocational School of Technical Sciences	15	5.1
Ataturk's Principles and History of Revolution	9	3.2
Faculty of Forestry	4	1.4

a Faculty of health sciences, Faculty of dentistry, Medical school and health care delivery vocational school.

### Data of academic research status of academicians

More than half of the participants (67.2%) reported that their academic research was negatively affected. Anxiety (47.9%), difficulty in concentration (41.0%), loss of motivation (36.9%), and not reaching academic fields of study (37.2%) were found to be the main factors that negatively affected academic research. It seems that more than half of the participants (69.3%) do not want the FWA to continue after the pandemic (Table 2).

**Table 2:** Academic reseraches of academicians duruing FWA (N=290).

Variables	n	%
**How flexible working affected the academic studies**		
Positive	95	32.8
Negative	195	67.2
**Positive factors**^a^		
Decrease in education workload	99	34.1
Decrease in administrative workload	24	8.3
More efficient use of time	112	38.6
Read more articles	66	22.8
Moving away from social dialogues that reduce the motivation to work	65	22.4
Less affected by climatic conditions	21	7.2
Increased rest and break opportunity	69	23.8
The increase in meetings and work on the internet	35	12.1
Feeling well due to the change of workplace	43	14.8
**Negative factors**^a^		
Difficulty in concentrating	119	41.0
Loss of motivation	107	36.9
Increase in housework	83	28.6
Anxiety	139	47.9
Being away from the work environment	57	19.7
Not getting permission from the ethics committee	36	12.4
Laboratories are closed	39	13.4
Not reaching academic fields of study	108	37.2
**Requesting continuation of flexible working**		
Yes	89	30.7
No	201	69.3

a Multiples of n because more than one option can be marked.

Of the academicians, 71% reported that they did not do any academic research during this period. It was determined that 40.5% of the academicians who reported having conducted academic research worked on review articles, 23.8% wrote research articles, 39.3% completed unfinished studies, and 34.5% read. Of these, 26.9% of the participants stated that they are working on COVID-19. Researchers working on COVID-19 reported that they mostly conducted research in the form of review articles (58.9%), descriptive (44.9%) methods, and cross-sectional (22.6%) methods. The rate of those conducting experimental research was 6.4% (Table 3).

**Table 3:** Academic research status of academicians during FWA.

Variables	n	%
**Conducting academic research**		
Yes	84	29.0
No	206	71.0
**Academic research type**^a^**(n=84)**		
Writing a research paper	20	23.8
Writing a review article	34	40.5
Refereeing / editing	22	26.2
Writing the book / chapter	16	19.0
Reading academic papers	29	34.5
Participating in online course	19	22.6
Completing unfinished academic studies	33	39.3
Conducting a graduate thesis	26	30.9
**Conducting academic research about COVID-19**		
Yes	78	26.9
No	212	73.1
**Academic research type about COVID-19**^a^**(n=78)**		
Descriptive	35	44.9
Cross-sectional	29	37.2
Experimental	5	6.4
Methodological	4	5.1
Case-control	9	11.5
Review article	46	58.9

a Multiples of n because more than one option can be marked.

### Correlated variables for academic research status of academicians

Table 4 shows the distribution and statistical results of some variables related to the academician's pursuit of academic research and how academic research was affected during FWA. It was observed that women did not conduct academic research at a higher rate than men (53.9% and 46.1%, p < 0.05, respectively). The student's t-test revealed that there was a statistically significant relationship between high CAS and not conducting academic research (p < 0.001).

**Table 4:** Distribution of some qualities of academicians on academic research status during FWA.

	Conducting academic research			How academic research was affected		
Variables	Yes n (%)	No n (%)	x^2^, t / p	Positive n (%)	Negative n (%)	x^2^, t / p
Gender, n (%)						
Woman	16 (19.1)	111 (53.9)	**5.780/0.016**	20 (21.1)	107 (54.9)	**29.682/0.000**
Man	68 (80.9)	95 (46.1)		75 (78.9)	88 (45.1)	
Age, n (%)						
29-34	32 (38.1)	73 (35.4)	1.317/0.105	26 (27.4)	79 (40.5)	10.067/0.064
35-44	27 (32.1)	74 (35.9)		42 (44.2)	59 (30.3)	
45-54	19 (22.6)	50 (24.3)		18 (18.9)	51 (26.2)	
55-64	6 (7.2)	9 (4.4)		9 (9.5)	6 (3.1)	
Marital Status, n (%)						
Single	20 (23.8)	55 (26.7)	0.260/0.610	27 (28.4)	48 (24.6)	0.483/0.487
Married	64 (76.2)	151 (73.3)		68 (71.6)	147 (75.4)	
Having a Child, n (%)						
Yes	70 (83.3)	150 (72.8)	3.605/0.058	70 (73.7)	150 (76.9)	0.366/0.545
No	14 (16.7)	56 (27.2)		25 (26.3)	45 (23.1)	
Faculty Type, n (%)						
Health^a^	34 (40.5)	73 (35.4)	38.100/0.050	4 (4.2)	103 (52.8)	**64.829/0.000**
Others	50 (59.5)	133 (64.6)		91 (95.8)	92 (47.2)	
Title, n (%)						
Prof. Dr.	4 (4.7)	18 (8.7)	45.013/0.312	5 (5.3)	17 (8.7)	23.035/0.050
Assoc. Dr.	13 (13.1)	11 (6.3)		20 (21.1)	4 (2.1)	
Dr. Lecturer	50 (61.9)	91 (44.2)		38 (40.0)	103 (52.8)	
Instructor	15 (17.9)	77 (36.4)		28 (29.5)	64 (32.8)	
Research Assistant	2 (2.4)	9 (4.4)		4 (4.2)	7 (3.6)	
Coronavirus Anxiety Score, mean ± SD	1.807 ± 1.314	2.664 ± 1.063	**-5.305/0.000**	1.174 ± 1.062	2.485 ± 1.192	**-9.093/0.000**

Values with p < 0.05 and p < 0.001 were shown in bold.

a Faculty of health sciences, Faculty of dentistry, Medical school and health care delivery vocational school.

It was found that the academic research of women was affected more negatively than that of men (54.9% and 45.1%, p < 0.001, respectively), and the research of academicians in health faculties was more negatively affected than those in other faculties (52.8% and 47.2%, p < 0.001, respectively). The student's t-test showed that those with high CAS reported the negative impact of academic research (p < 0.001) (Table 4).

### Correlated variables for academician coronavirus anxiety scores

It was concluded that the academician's total scores on the CAS exhibited normal distribution and that their mean score was 2.055 ± 1.304 (range: 0–4). The distribution and statistical results of some of the variables related to the CAS among academicians are provided in Table 5. CAS in women was higher than men (respectively: 14.055 ± 4.824; 7.337 ± 6.156), and those working in health faculties (respectively: 15.878 ± 3.793; 7.005 ± 5.473) higher (p < 0.001). Anova test showed that the youngest group reported the highest CAS (p < 0.001).

**Table 5:** Distribution of CAS among academicians during FWA.

Variables	CAS^a^	t, F / p
Gender, mean ± SD		
Women	14.055 ± 4.824	**10.112/0.002**
Men	7.337 ± 6.156	
Age, mean ± SD		
29-34	15.4667 ± 5.370	**10.690/0.000**
35-44	11.4952 ± 5.977	
45-54	11.0000 ± 6.247	
55-64	7.7525 ± 6.542	
Marital status, mean ± SD		
Single	10.786 ± 4.960	2.342/0.102
Married	9.753 ± 6.918	
Having a Child, mean ± SD		
Yes	10.009 ± 6.672	-1252 / 0.174
No	11.128 ± 5.992	
Faculty type, mean ± SD		
Health area^b^	15.878 ± 3.793	**14.812/0.000**
Others	7.005 ± 5.473	
Title, mean ± SD		
Prof. Dr. (22)	8.772 ± 5.789	6.772/0.058
Assoc. Dr. (24)	5.958 ± 5.465	
Dr. Lecturer (141)	9.280 ± 6.784	
Instructor (92)	8.293 ±6.358	
Research Assistant (11)	9.454 ± 6.055	

Values with p < 0.05 and p < 0.001 were shown in bold.

a Coronavirus Anxiety Scale.

b Faculty of health sciences, Faculty of dentistry, Medical school and health care delivery vocational school.

## Discussion

This was a cross-sectional study conducted in a state university in Kahramanmaras with 290 academicians. The present study was the first in Turkey to assess the effect of the COVID-19 pandemic on the academic research status of academicians’ and their CAS score, and to identify related variables.

The study revealed that most academics have not conducted academic research during the post-pandemic FWA, and the academic research of most academics has been negatively affected. This result is important in terms of revealing the negative impact of COVID-19 on academic knowledge generation.

The findings of this study showed that during this period, women academicians conducted a lower percentage of academic research than men, and much of their research was negatively affected. It has been reported that female academics could not conduct sufficient research in the pre-pandemic period due to the difficulties they face such as male-dominated institutional cultures, lack of female mentors, and gendered domestic workforce times.^20^^,^^24^ These findings have been reported in another study, which revealed that the productivity and scientific outcomes of female academics were disproportionately affected during COVID-19.^20^ In another study, they compared 37.531 articles published in 2019 and 1179 medical COVID-19 articles published in 2020. According to the evaluation, it was determined that the number of women academics being the general, first, and last authors of articles during the COVID-19 pandemic was negatively affected compared to 2019.^25^ This unfavourable situation of the research of women academics can be explained by the increased workload of women during FWA. One study found that during the COVID-19 outbreak in the US, UK, and Germany, women spent more time than men on childcare and home-schooling.^26^

The academic research of participants working in health faculties was affected more negatively. This finding can be explained by the higher CAS score of academicians working in health faculties, and the inadequate research conducted by academics with high anxiety levels. In addition, findings that negatively affect the research of the participants include ‘inability to access the field of study’, and ‘not getting permission from the ethics committee’. These problems may arise due to the FWA of academicians on ethical committees and the researchers in health sciences.

In this study, concentration difficulties and loss of motivation were the main reasons that negatively affected academic research during FWA. Other studies have found that flexible working eliminates people's interactions with their colleagues and negatively affects work efficiency due to difficulties in time management.^27^^,^^28^ Similarly, in a study examining the effects of flexible working of academic staff during COVID-19 pandemic, a significant proportion of the participants reported that working at home increased distraction and hindered focus compared to working in the office.^29^ Based on these findings, it can be said that FWA negatively affects academic research for various reasons.

In addition, in our study, the participants reported that they could not do enough work owing to the access barrier to academic study sites and due to anxiety. This situation was likely caused by access barriers that emerged during the COVID-19 pandemic due to curfews and FWA in many institutions. Especially in our study, a significant portion of the participants work in health faculties and academic studies in this field are mostly carried out in institutions affiliated to the Ministry of Health. The number of academic studies may have been limited due to the possibility of coronavirus transmission in academic study areas. This hypothesis supports another finding of our study. In our study, it was determined that the academicians who reported that they were doing academic work primarily completed unfinished academic studies and read academic articles. The low number of new academic studies that were initiated can be explained by this situation. Notably, participants indicated anxiety as one of the reasons that prevent them from doing academic work. In addition to FWA, it is thought that the mental state created by the pandemic period negatively affects the number of academic studies.

A positive association was found between the CAS score and female gender. Similar findings have been reported in other research. In a study involving 69 academic staff in Africa, it was reported that the coronavirus anxiety score was higher in women.^30^ In another study conducted by Rakhmanov and Dane (2020) with university students in Africa, it was seen that female students have higher coronavirus anxiety scores than male students.^15^ Hosseinzadeh-Shanjani et al. (2020), in their study including 200 healthcare workers in Iran, found that female healthcare workers had higher COVID-19 anxiety scores than men.^16^ General anxiety studies conducted in the past indicate that the anxiety rates of women are higher.^15^, ^16^, ^17^ This can be explained by women's greater vulnerability to stressful events, their intense emotional response to stress, and the bio-psychological characteristics of women.

Academicians working in faculties in the field of health had significantly higher CAS score than those working in other fields. This can be explained by the higher awareness of academicians working in the field of coronavirus. In addition to this, physicians and dentists working in the hospital were among the academicians who participated in our study, which may have resulted in higher anxiety scores. A study conducted in Iran found that the anxiety levels of healthcare workers in the COVID-19 pandemic are higher than other professions and the general population.^31^ Based on these findings, it can be said the coronavirus anxiety levels of clinicians and academic health professionals are higher than the general population.

Although COVID-19 infections are known to cause significantly higher morbidity and mortality in the older group than the younger age group, studies have found higher anxiety scores in the younger age group.^32^, ^33^, ^34^ Similarly, our results indicate that the prevalence rates for high coronavirus anxiety scores on CAS were highest amongst those age between 29 and 34 years, and lowest amongst those between 55 and 64 years. While younger, more resilient, and risk-averse individuals may experience increased anxiety during this pandemic, this relationship reverses with age, such that older, more resilient, and risk-averse individuals experience less anxiety during the COVID-19 outbreak.^35^ Age provides opportunities to develop resistance due to exposure to multiple and different stressors over time, resulting in better emotional management and lower anxiety.^36^ These findings suggest that there is a negative correlation between age and coronavirus anxiety symptoms.

A comprehensive study conducted in China reported that about 35% of people were psychologically affected by the COVID-19 pandemic. Among these psychological effects, a high rate of anxiety was revealed.^11^ A study conducted in Turkey has reported widespread anxiety due to COVID-19.^12^ High anxiety level affect the person negatively in many ways. Studies have determined that high anxiety levels negatively affect students' academic achievement.^13^^,^^14^ However, no study has examined the effect of coronavirus anxiety on academicians. In the present study, we found that the academicians with high CAS scores conducted a lower percentage of academic research, and much of their research was negatively affected. This finding is important in terms of revealing the effect of coronavirus anxiety on academics.

### Limitations

This research had some limitations. First, the participants were contacted via their e-mail addresses and many academicians may not have seen the research invitation e-mail. This directly affected the sample size. Second, our sample is only representative of the sampled participants and cannot be generalized. Third, research data on anxiety were collected using self-report and data collection tools. The findings depend on the reliability and sensitivity of the data collection tools and cannot be generalized to cases with clinical diagnostic criteria. Fourth, the participants were not asked about whether they or their relatives had been infected with the COVID-19 virus.

## Conclusions

The study revealed that most of the academicians did not conduct academic research during this period and academic research was largely negatively affected. Furthermore, women and those with higher coronavirus anxiety scores had higher rates of not conducting research. Additionally, the research of women, participants working in health faculties, and participants with high coronavirus anxiety scores was more negatively affected. Women, those working in healthcare faculties, and the youngest group reported the highest anxiety scores.

Online coronavirus anxiety management programs are recommended to improve anxiety and coping strategies as well as prevent further psychological consequences. Also, as this is the first survey on the psychological impact of COVID-19 on Turkish academics, these results can be used as a basis for investigating coronavirus anxiety and the extent of its effects.

Further studies are required to make longitudinal evaluations of psychiatric disorders such as anxiety. More longitudinal prospective studies using a large population and different time series are recommended to validate the results of this study and provide a more comprehensive understanding of this topic. It may be suggested that researchers conducting similar studies should plan to overcome the limitations of our study. Since the online questionnaires are sent via e-mail in the flexible working arrangement, a significant portion of the academicians may not have seen the e-mail and were not included in the study. Thus, data should be collected using different interview methods in future studies. In addition, a question on the participants’ history of infection with the coronavirus should be added to the questionnaire as it may affect their academic studies and coronavirus anxiety.

## Source of funding

This research did not receive any specific grant from funding agencies in the public, commercial, or not-for-profit sectors.

## Conflict of interest

The authors have no conflict of interest to declare.

## Ethical approval

In order to conduct the study, approval was obtained from the Ethics Committee (KSU 2020-13; 243) and the KSU Rectorate dated 24.06.2020. The research followed the principles of the Helsinki Declaration. Written informed consent was obtained from all participants before starting the questionnaire.

## Authors' contributions

DA and her co-author ŞD conceptualised and designed the study; provided research materials; collected, analysed and interpreted data; wrote the manuscript; and critically reviewed the final draft. All authors have critically reviewed and approved the final draft and are responsible for the content and similarity index of the manuscript.
